# Comparison of the serum metabolic signatures based on ^1^H NMR between patients and a rat model of deep vein thrombosis

**DOI:** 10.1038/s41598-018-26124-x

**Published:** 2018-05-18

**Authors:** Jie Cao, Qian-qian Jin, Gui-ming Wang, Hong-lin Dong, Yong-ming Feng, Jun-sheng Tian, Ke-ming Yun, Ying-yuan Wang, Jun-hong Sun

**Affiliations:** 1 School of Forensic Medicine, Shanxi Medical University, Taiyuan, 030001 P. R. China; 2 Department of Vascular Surgerey, First Hospital of Shanxi Medical University, Taiyuan, 030001 P. R. China; 3 Department of Vascular Surgerey, Second Hospital of Shanxi Medical University, Taiyuan, 030009 P. R. China; 4 Modern Research Center for Traditional Chinese Medicine, Shanxi University, Taiyuan, 030006 P. R. China

## Abstract

Deep vein thrombosis (DVT) and pulmonary embolism (PE) have high morbidity, reduce quality of life, and can cause death. Biomarkers or genetic risk factors have not been identified in patients with DVT. In present study, serum of 61 patients suffering from DVT and a rat DVT model (n = 10) were assayed by a proton nuclear magnetic resonance (^1^H NMR) metabolomics technique combing with multivariate statistical analysis to identify the metabolites. The MetPA platform was used to identify differences in the metabolic pathways between the rat model and patients. The metabolomics results discovered that 11 different metabolites in rats and 20 different metabolites in DVT patients. Seven metabolites both altered in the rats and patients. Moreover, we observed changes in the metabolic pathways, including carbohydrate metabolism, lipid metabolism, and amino acid metabolism that were induced immediately by the thrombosis. Pathway of aminoacyl-tRNA biosynthesis perturbed only in the patients which was associated with the genetic risk factor of DVT. The study demonstrated that serum ^1^H NMR metabolomics can be used to diagnose DVT in the clinic. The altered pathways related to thrombosis and genetics will provide a foundation and new strategies for understanding the pathological mechanism and pharmacological targets of DVT.

## Introduction

Deep vein thrombosis (DVT) is a condition in which a blood clot forms in a deep vein, most commonly in the lower extremities. The most severe complication of DVT is a pulmonary embolism (PE), which may lead to chest pain, dyspnea, or death^1^. It is the third most common vascular disease after myocardial infarction and ischemic stroke. The morbidity of DVT is about 1–3/1000 individuals per year in industrialized countries^2–5^, and it is expected to increase in the coming decades due to aging populations worldwide and an increase in the frequencies of DVT risk factors^6,7^.

DVT is a multifactorial disorder resulting from the interaction between an array of acquired and genetic risk factors. The main inherited thrombophilias include plasma deficiencies of natural anticoagulants antithrombin and proteins C and S; the gain-of-function mutations factor V Leiden and prothrombin G20210A; some dysfibrinogenemias, and high plasma levels of coagulation factor VIII^8,9^. According to these genetic risk factors, only about 40% of all previously unexplained episodes of venous thromboembolism (VTE) can be explained. Therefore, it is necessary to identify additional potential genetic risk factors to understand the mechanism of DVT for timely treatment, improve the patient prognosis, and reduce mortality.

Early diagnosis of DVT is also important. The clinical diagnostic strategy for DVT depends mainly on a clinical assessment, determination of the D-dimer level, and compression ultrasonography. However, the diagnostic process for acute DVT is challenging due to a lack of specific clinical symptoms. The plasma D-dimer level has high sensitivity and its absence may help exclude DVT. However, D-dimer is susceptible to changes due to cancer, surgery, and other factors, so specificity and positive predictive value are very low^10^. The same situation surrounds debates on other VTE biomarkers, such as P-selectin, microparticles, and C-reactive protein^11^. Thus, more appropriate biomarkers are needed to make a proper risk assessment of VTE and its recurrence to avoid invasive procedures.

The development of systems biology, such as genomics, proteomics, and metabolomics, has provided a more effective way to study the pathogenesis of diseases and search for new diagnostic biomarkers^12^. As an important component of systems biology, metabolomics has been widely applied to study diagnostic and prognostic biomarkers related to disease and the pathogenesis of disease by detecting endogenous small compounds in biological samples^13^.

Metabolomics is a rapidly evolving field that holds promise to provide insight into genotype–phenotype relationships in cancers, diabetes, and other complex diseases. The MetPA tool and Metscape links metabolite data to metabolic pathways. The potential genetic risk factors related to metabolites, genes, or transcription could explain the mechanism of complex diseases, such as DVT.

In the present study, Sprague–Dawley rats without genetic abnormalities were used to establish a DVT rat model. In addition, patients from several hospitals in Taiyuan and Shanxi with a confirmed first episode of unprovoked DVT were recruited. The metabolic profiles of the serum samples from the DVT rat model and patients were investigated using high-resolution proton nuclear magnetic resonance (^1^H NMR) spectroscopy. The aim of this study was to identify the alterations in metabolites at the molecular level in rats and patients with DVT and to develop a biomarker panel that could be used to screen for the disease. More importantly, we attempted to distinguish the pathways associated with genetic or acquired risk factors by comparing the altered pathways between rats and humans.

## Results

### ^1^H NMR spectra of individual serum in the DVT rat model

**Figure 1:**
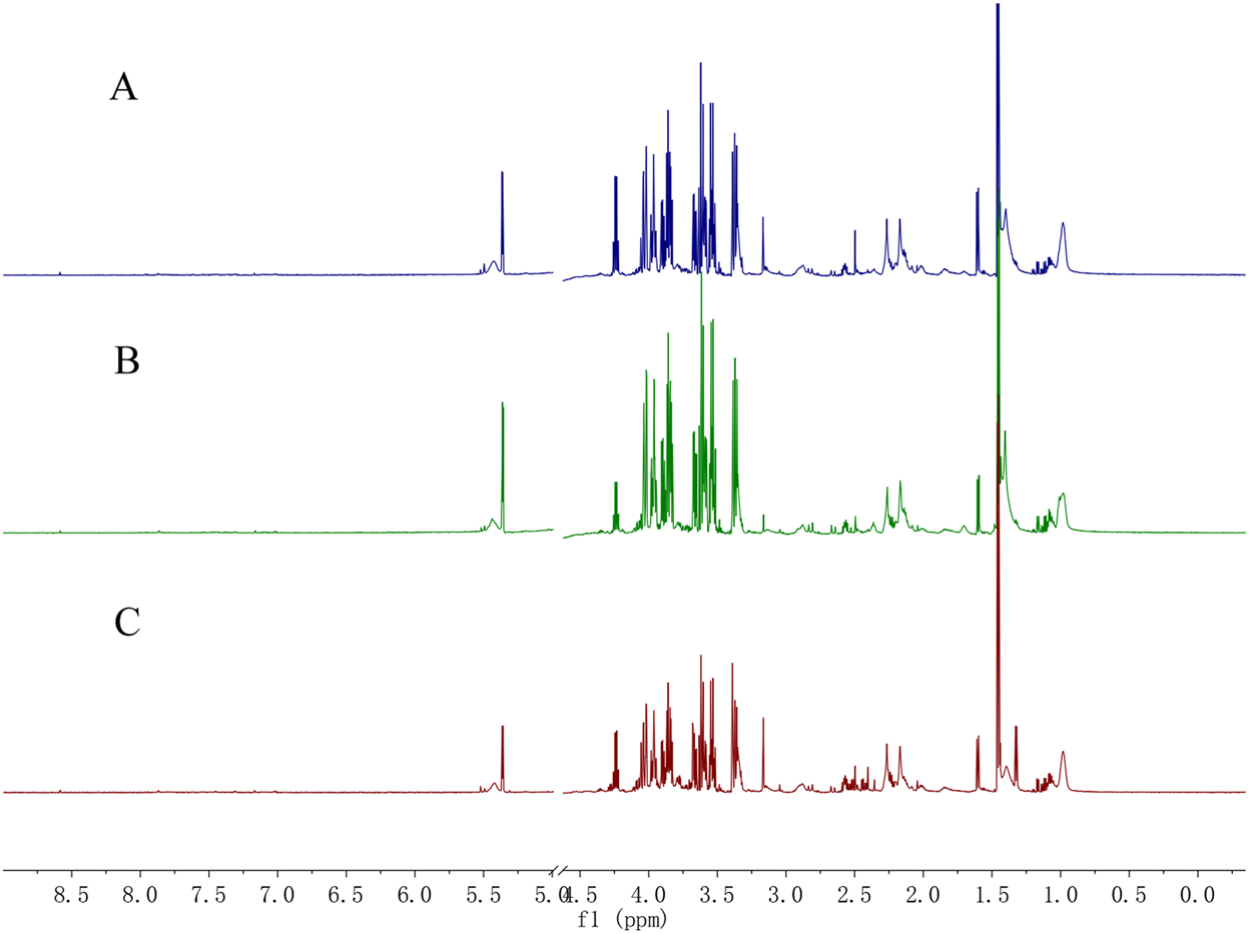
Representative proton nuclear magnetic resonance (^1^H NMR) spectra of the rat serum in the different groups. (**A**) Deep vein thrombosis (DVT) group; (**B**) sham group; and (**C**) control group.

Representative ^1^H NMR spectra of serum samples collected from the DVT, sham, and control groups are displayed in Fig. 1. The assignments of endogenous metabolites in the ^1^H NMR spectra were based on comparing chemical shifts and multiplicities of peaks to the 600-MHz Chenomx^TM^ database of small molecules (Chenomx NMR Suite 8.0, Inc., Edmonton, AB, Canada) and published refs^14–16^. A number of endogenous metabolites were identified from the spectra, such as acetate, lactate, alanine, leucine, glucose, valine, and pyruvate.

### Differences in the serum metabolomics of the rat model

**Figure 2:**
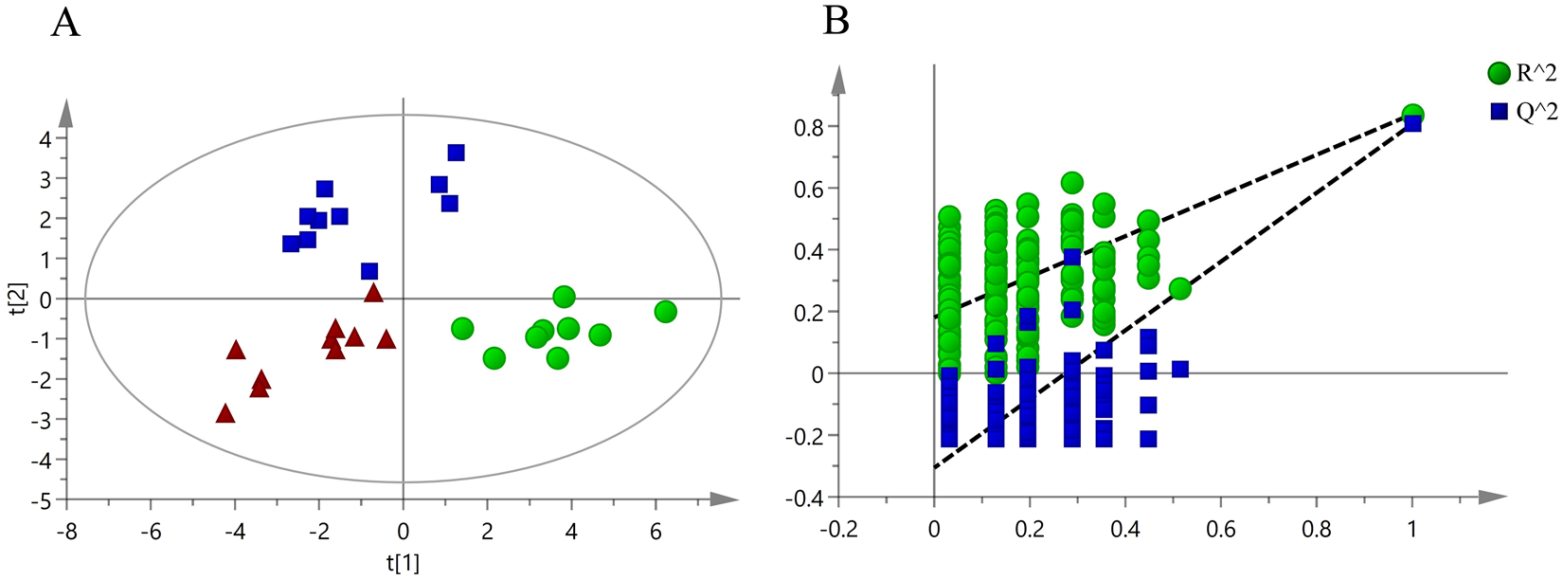
Multivariate analysis of serum samples from the control, sham, and DVT rats. (**A**) Partial least squares discrimination analysis (PLS-DA) score plot derived from all of the ^1^H NMR spectra of the sera collected from rats in the DVT group (⦁), sham group (▪), and control group (▴). (**B**) The PLS-DA validation plots (permutation times: 200) for all samples including the DVT, sham, and control groups.

We observed differences in the metabolomics by comparing the resulting integral data derived from the spectra of the rat serum collected from the three groups. Multivariate statistics were performed to analyze the NMR spectral information. As shown in Fig. 2, partial least squares discrimination analysis (PLS-DA)-based profiling was used to explore the intrinsic differences among the groups. Each point represents the serum metabolome of one subject, and the distance between data points reflects the scale of their metabolic differences (Fig. 2A). The PLS-DA model (R^2^X = 0.422, R^2^Y = 0.794, Q^2^ = 0.693) showed clear distinctions among the control, sham, and DVT groups. Furthermore, the permutation test (200 times) and cross-validated residuals analysis of variance (CV-ANOVA) (p < 0.05) showed that the constructed PLS-DA model was positive and valid (Fig. 2B). All of these results indicate the differences among the three groups.

**Figure 3:**
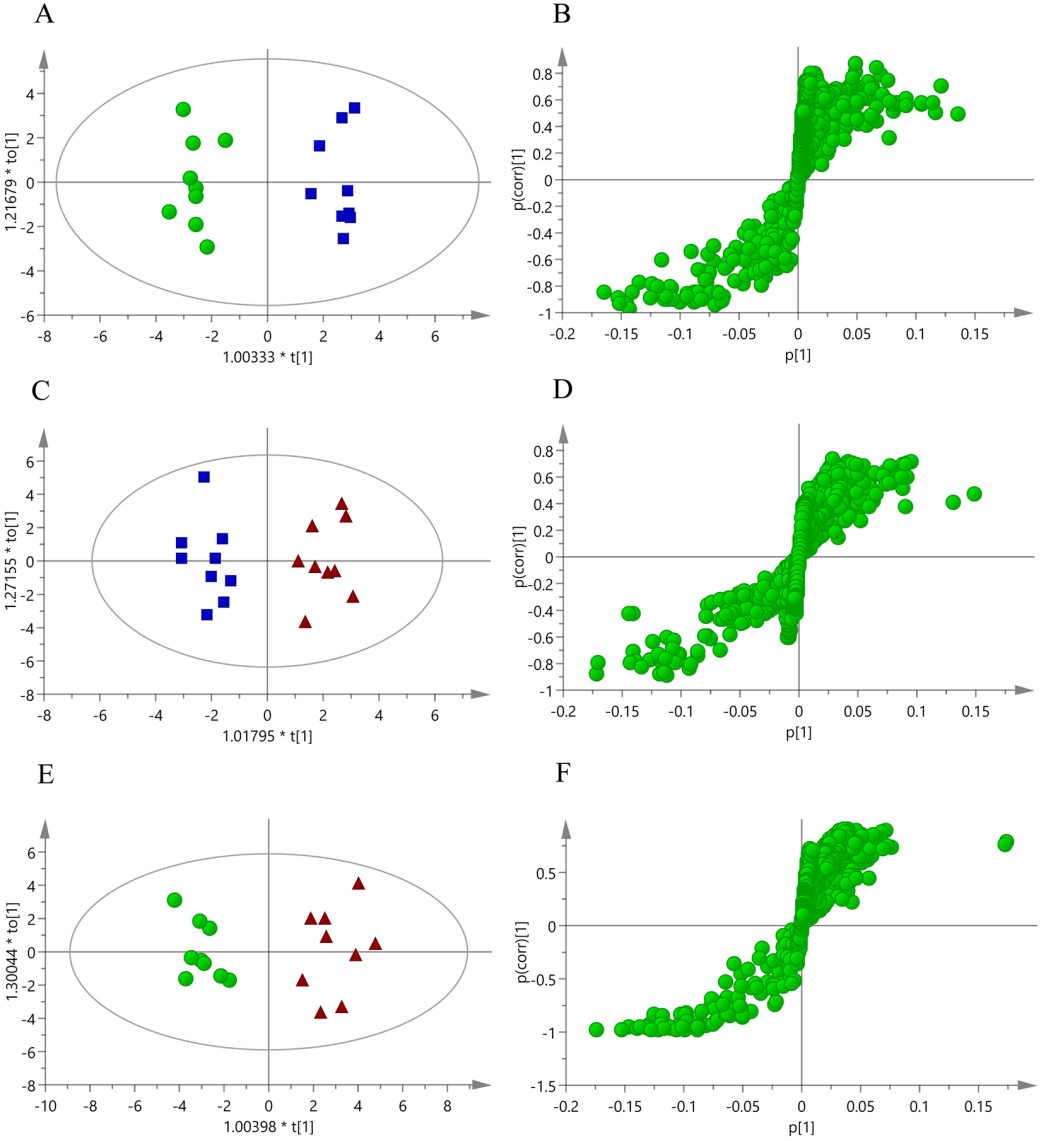
Multivariate analysis of serum samples from control, sham, and DVT rats. (**A**) The orthogonal partial least squares discrimination analysis (OPLS-DA) score plot derived from all of the ^1^H NMR spectra of the serum in the sham (▪) and control groups (▴). (**B**) Corresponding S-plot between the sham and control groups. (**C**) The OPLS-DA score plot between the DVT (⦁) and control groups (▴). (**D**) Corresponding S-plot between the DVT and control groups. (**E**) The OPLS-DA score plot between the DVT (⦁) and sham groups (▴). (**F**) Corresponding S-plot between the DVT and sham groups.

To identify the metabolites that changed and considering the high information content and complexity of the spectra, an orthogonal partial least squares discrimination analysis (OPLS-DA) approach was applied to reveal any subtle changes due to DVT or the operation (shown in Fig. 3). The supervised OPLS-DA model developed a better separation into two clusters and contributed to the discovery of biomarkers. A pairwise analysis also exhibited a well-segregated and gathered form of the OPLS-DA score plots. The results demonstrated robust metabolic differences between the DVT and sham (Fig. 3A), the DVT and control (Fig. 3C), and the sham and control groups (Fig. 3E). The permutation tests (200 times) and CV-ANOVA (p < 0.001) results are shown in Table 1, indicating that the OPLS-DA model of the three groups was statistically sound. Each point in the corresponding S-plot of the OPLS-DA represents a spectral region, which is a metabolite marker (Fig. 3B,D,F). A few of these markers were verified from the same metabolite. The points at the ends of the S-plot curve indicate higher contributions to the classification.

**Table 1:** Orthogonal partial least squares discrimination analysis parameters from the rat sera samples.

	R^2^X	R^2^Y	Q^2^	CV-ANOVA
Control *vs* sham	0.463	0.963	0.864	1.523E-005
Control *vs* DVT	0.548	0.919	0.786	0.00027
Sham *vs* DVT	0.393	0.919	0.748	0.00754

**Figure 4:**
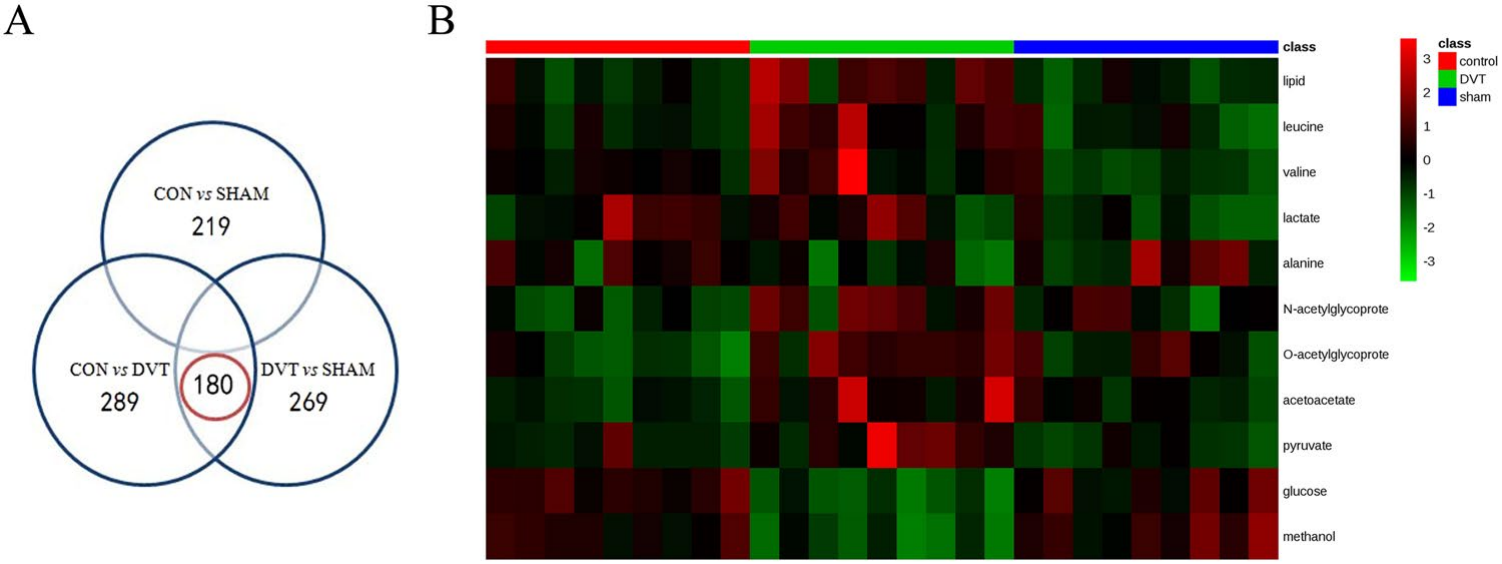
Venn diagram and heatmap plot from the comparisons of DVT, sham, and control groups. (**A**) Venn diagram of different spectral regions. The numbers in the overlapping areas (encircled in red) represent different spectral regions between the DVT group and the other two groups, but not between the control and sham groups. (**B**) Heatmap plot of the different metabolites. Red color indicates a higher level and green color indicates a lower level.

In the present study, metabolites with significant differences were screened according to their corresponding variable importance in the projection (VIP) values of these OPLS-DA models (Table 2). As shown in Fig. 4A, 219, 289, and 269 spectral regions were found in the control *vs*. sham group, control *vs*. DVT group, and sham *vs*. DVT group, respectively. To eliminate the effects of the surgical operation, 180 spectral regions in overlapping areas (red circle in Fig. 4A), indicated differences between the DVT group and the other groups, but no difference between the control and sham groups.

**Table 2:** Key metabolites to discriminate serum from rats with a deep vein thrombosis (DVT) from the sham and control groups.

No	Metabolites	KEGG IDs	Chemical shift^a^	DVT *vs* control	
				p values^b^	VIP^c^
1	lipid	NA	0.88(m), 1.27 (m)	↑^*^	4.05
2	leucine	C00123	0.95 (dd)	↑^*^	1.65
3	valine	C00183	0.98 (d), 1.04 (d)	↑^*^	1.28
4	lactate	C00186	1.32 (d), 4.12 (q)	↓^**^	5.18
5	alanine	C00041	1.46 (d)	↓^**^	2.45
6	NAC	NA	2.04 (s)	↑^*^	1.18
7	OAC	NA	2.14 (s)	↑^*^	1.16
8	acetoacetate	C00164	2.28 (s)	↑^**^	1.13
9	pyruvate	C00022	2.37 (s)	↑^*^	2.16
10	glucose	C00031	3.25 (t), 3.42 (m), 55(dd), 3.72 (m), 3.90 (dd), 5.24(d)	↓^***^	6.97
11	methanol	C00132	3.39 (s)	↓^***^	4.69

^a^S, singlet; d, double; t, triple; q, quartet; m, multiplet; dd, double doublet. ^b^P-values were derived from two-tailed Student’s *t*- test: ^*^p < 0.05, ^**^p < 0.01, ^***^p < 0.001. Metabolites marked with ↑ increased compared with the respective group; metabolites marked with ↓ decreased compared to the respective group. ^c^Variable importance in the projection (VIP) value was obtained from OPLS-DA with a threshold of 1.0. NAC: N-acetylglycoproteins, OAC: O-acetylglycoproteins.

A *t*-test was performed to further verify these different spectral regions. The results showed that 124 of 180 spectral regions were significantly different (p < 0.05), and these spectral regions were assigned to 11 metabolites based on the Chenomx^TM^ database of small molecules (Table 2). The 11 metabolites could be used to differentiate DVT rats from the sham and control groups. The specific changing trends of higher levels of lipid, leucine, valine, N-acetylglycoproteins, O-acetylglycoproteins, acetoacetate, and pyruvate, and lower levels of lactate, alanine, glucose, and methanol are shown in Table 2. Furthermore, a heatmap plot, in which green represents a low level and red represents a high level, was constructed, from which we observed the trends visually (Fig. 4B).

### ^1^H NMR spectra of individual serum samples in patients with a DVT

**Figure 5:**
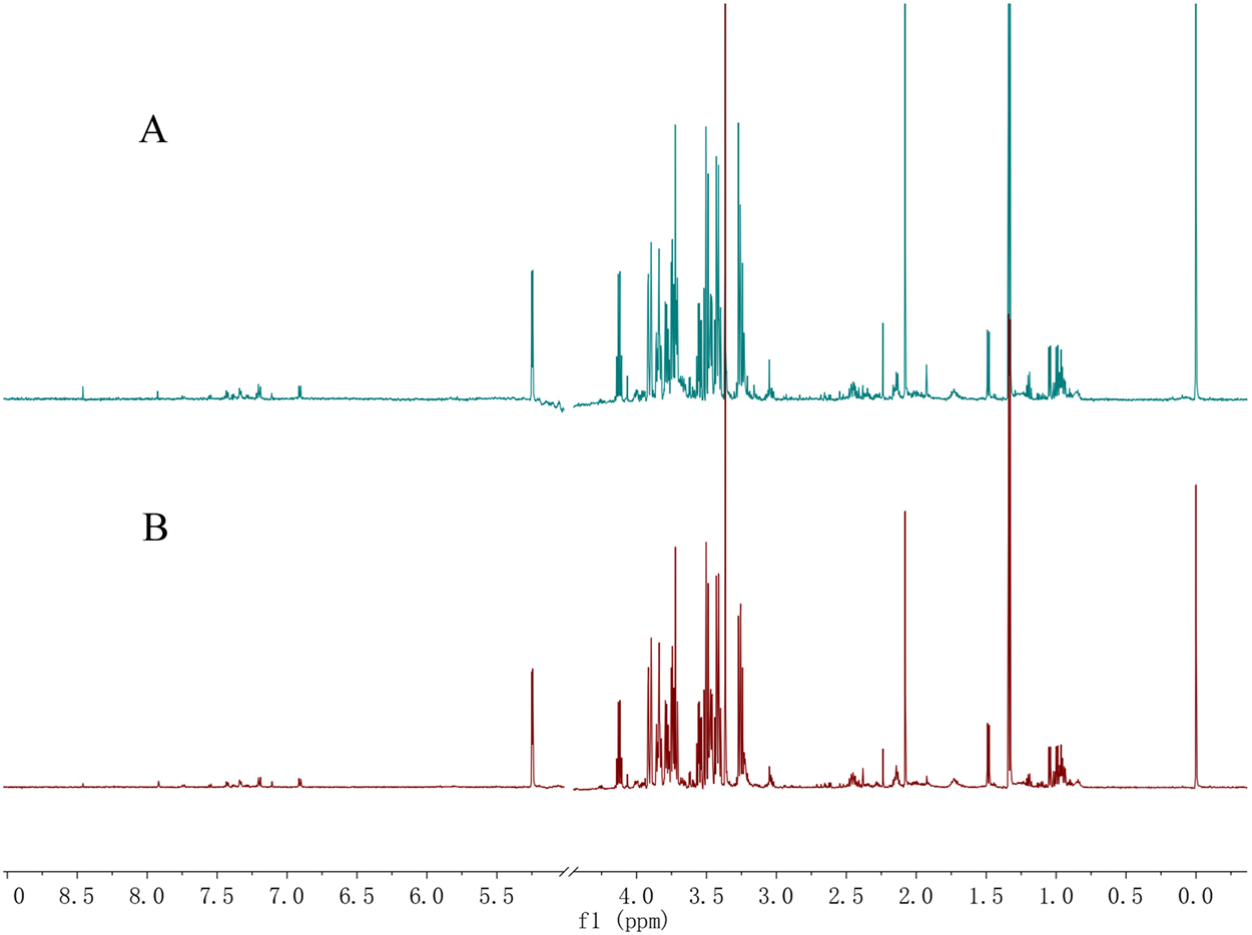
Representative ^1^H NMR spectra of individual serum samples in the different groups. (**A**) DVT patient group and (**B**) healthy control group.

According to the clinical diagnosis, 61 unprovoked patients (30 males and 31 females) had a DVT, and age- and sex-matched healthy control individuals were recruited from hospitals in Taiyuan and Shanxi. Representative ^1^H NMR spectra of serum samples collected from patients and healthy individuals are shown in Fig. 5. The assignments of endogenous metabolites in the ^1^H NMR spectra were consistent with those detected in the rats.

### Differences in the metabolomics analysis of serum in patients with a DVT

**Figure 6:**
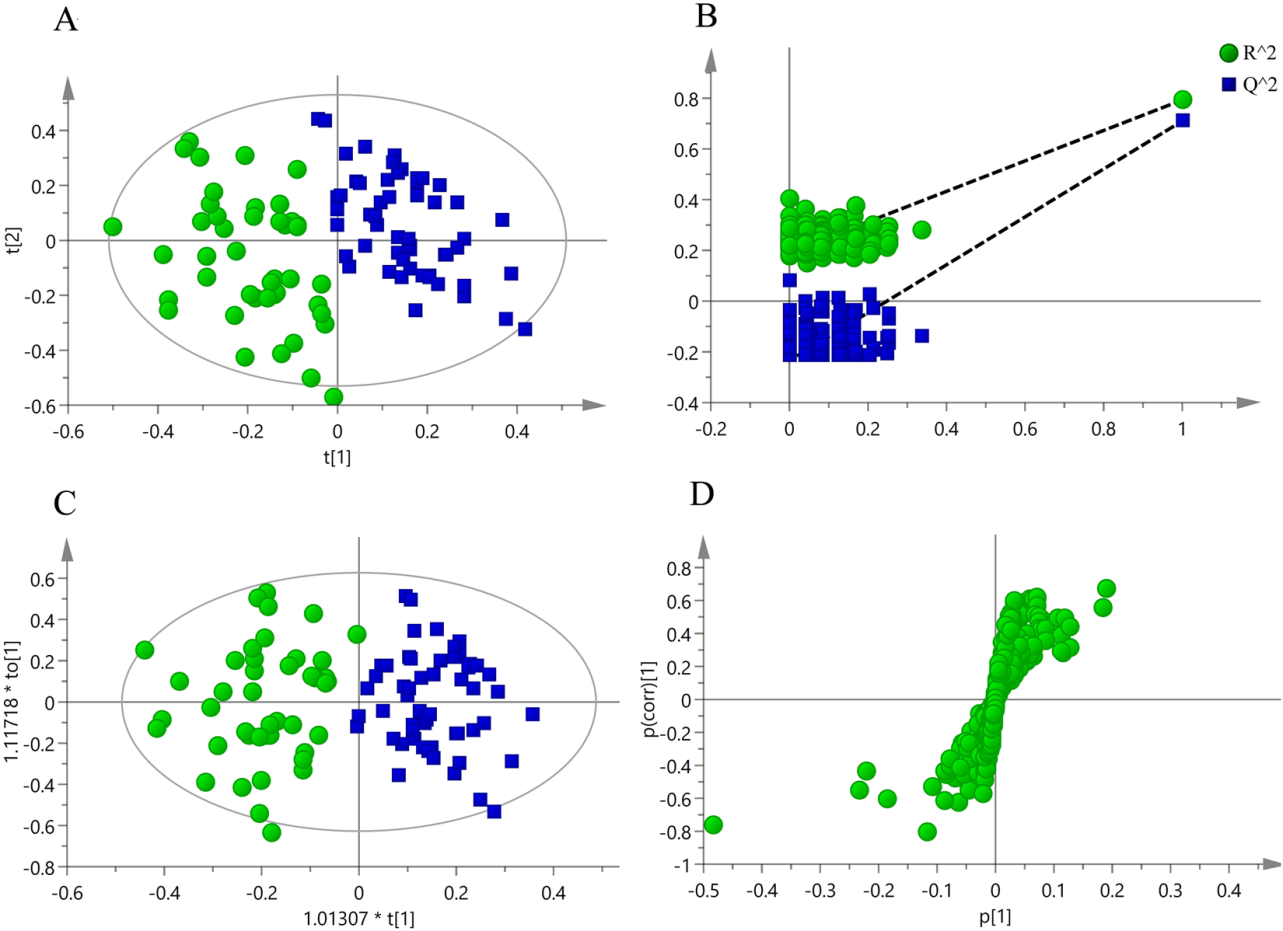
Multivariate analysis of serum samples from DVT patients and healthy individuals. (**A**) PLS-DA score plot derived from ^1^H NMR spectra of serum from DVT patients (⦁) and healthy individuals (▪). (**B**) The PLS-DA validation plot (permutation times: 200) for all samples including DVT patients and healthy individuals. (**C**) The OPLS-DA score plot between DVT patients (⦁) and healthy individuals (▪). (**D**) Corresponding S-plot between DVT patients and healthy individuals.

As shown in Fig. 6, the data analysis using supervised multivariate analysis via PLS-DA distinguished the patients from the healthy individuals. The samples from the two groups were separated and classified into two distinct clusters presented in the score plot (Fig. 6A). The model parameters (R^2^X = 0.26, R^2^Y = 0.793, Q^2^ = 0.710; p-value of CV ANOVA <0.001) and the validated model (permutation test, 200 times) demonstrated that the PLS-DA model was positive and valid (Fig. 6B). All of these results indicated a difference between the serum samples of patients with a DVT and healthy individuals.

The OPLS-DA analysis was performed to discern the effects of disease on the patients and healthy individuals. The OPLS-DA score plot (R^2^X = 0.26, R^2^Y = 0.793, Q^2^ = 0.737; p-value of CV-ANOVA <0.001) also showed a clear separation between the DVT patients and the healthy controls (Fig. 6C). The corresponding S-plot (Fig. 6D) and VIP values suggested that 235 different spectral regions existed between the DVT patients and healthy controls.

**Figure 7:**
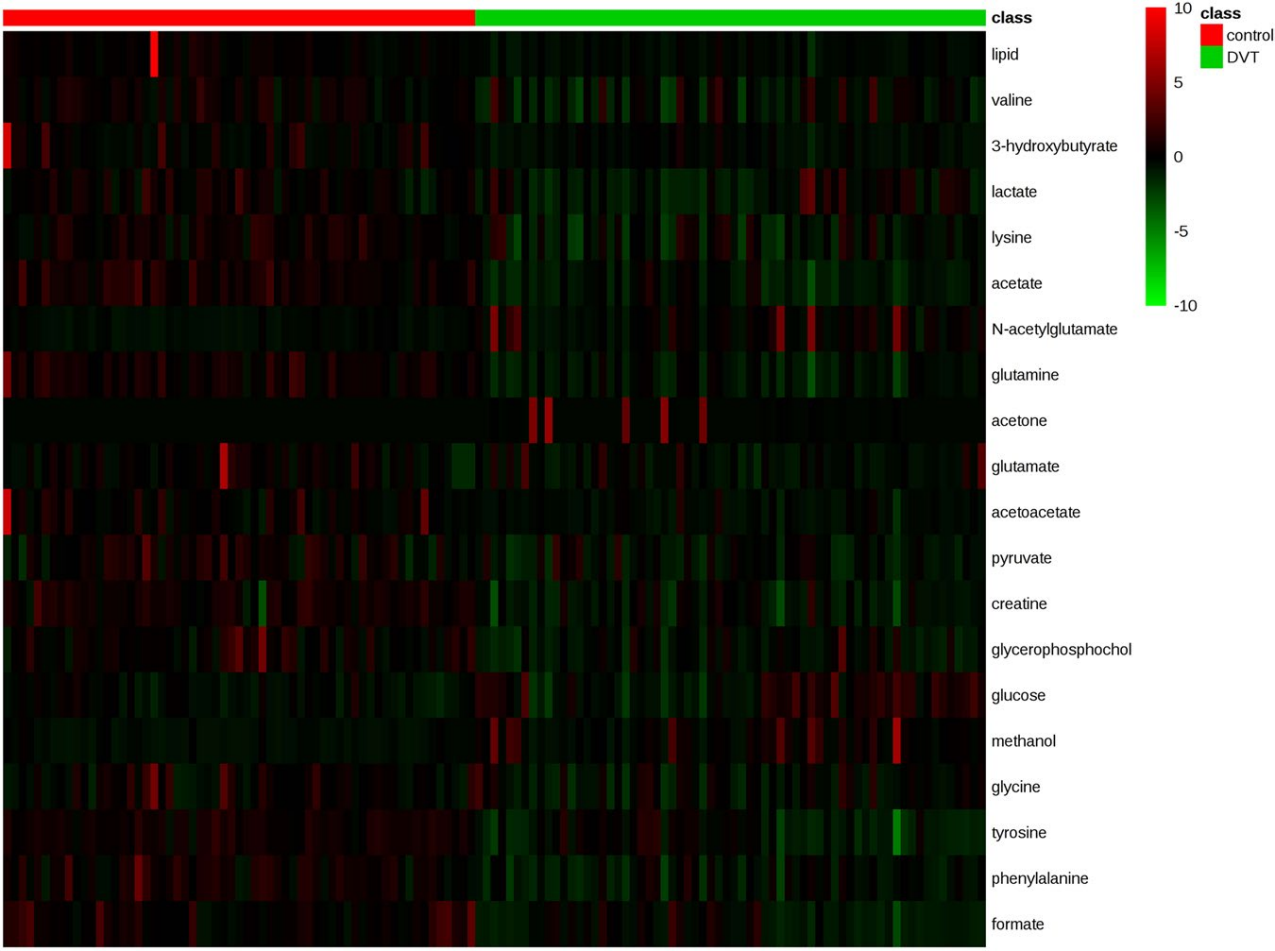
Heatmap plot between patients with DVT and healthy individuals. Red color indicates a higher level and green color indicates a lower level.

Student’s *t*-test was also performed to uncover the spectral regions. The results showed that 157 out of 235 spectral regions were significantly different (p < 0.05), which were assigned to 20 metabolites. The serum samples of DVT patients showed higher levels of lipids, valine, 3-hydroxybutyrate (3-HB), lactate, lysine, acetate, glutamine, acetoacetate, pyruvate, creatine, glycerophosphocholine, glycine, tyrosine, phenylalanine, and formate, but lower levels of N-acetylglutamate, acetone, glutamate, glucose, and methanol compared with healthy individuals (Table 3). A heatmap plot with different colors, in which green indicated a low level and red was a high level, was constructed, from which the trends could be observed (Fig. 7).

**Table 3:** Key metabolites responsible for discriminating serum from DVT patients and healthy individuals.

No.	Metabolites	KEGG IDs	Shift chemical	DVT *vs* control	
				p values^b^	VIP
1	^*^lipid	NA	0.88 (m), 1.29 (m)	↑^***^	1.38
2	^*^valine	C00183	0.98 (d), 1.04 (d)	↑^***^	1.30
3	3-HB	C01089	1.19 (d)	↑^***^	1.82
4	^*^lactate	C00186	1.32 (d), 4.12 (d)	↑^**^	5.20
5	lysine	C00047	1.72 (m)	↑^***^	1.02
6	acetate	C00033	1.91 (s)	↑^***^	3.14
7	NAG	C00624	2.08 (s)	↓^***^	7.06
8	glutamine	C00064	2.14 (m), 2.41 (m)	↑^***^	1.27
9	acetone	C00207	2.24 (s)	↓^*^	4.98
10	glutamate	C00217	2.34 (m)	↓^*^	1.68
11	^*^acetoacetate	C00164	2.28 (s)	↑^***^	1.53
12	^*^pyruvate	C00022	2.38 (s)	↑^***^	1.75
13	creatine	C00300	3.06 (s), 3.94 (s)	↑^***^	1.05
14	GPC	C00670	3.23 (s)	↑^***^	3.12
15	^*^glucose	C00031	3.26(t),3.41(m),3.56(dd), 3.90 (dd), 5.25(d)	↓^***^	3.59
16	^*^methanol	C00132	3.37 (s)	↓^***^	20.94
17	glycine	C00037	3.57 (s)	↑^**^	1.06
18	tyrosine	C00082	6.90 (d), 7.22 (d)	↑^***^	1.40
19	phenylalanine	C00079	7.32 (m)	↑^***^	1.45
20	formate	C00058	8.46 (s)	↑^***^	1.38

^*^Represents the metabolites that changed in DVT rats and patients. 3-HB, 3-hydroxybutyrate; GPC, glycerophosphocholine; NAG, N-acetylglutamate.

### Diagnostic test to evaluate the different metabolites

A receiver operating characteristic (ROC) curve analysis is a popular method to evaluate the accuracy of a medical diagnostic system. In the present study, a biomarker model consisting of the 20 metabolites from DVT patients was constructed by MetPA. The ROC curve analysis was used to evaluate the area under the curve (AUC) value of the model for the 20 metabolites. The AUC value was 0.986 (Figure S1A), indicating more effective sensitivity and specificity. A clear separation was observed between the patients and healthy controls in the probability view (Figure S1B). The average accuracy based on 100 cross validations was 0.939 (Figure S1C) in this study. The permutation test results (p < 0.001, Figure S1D) for the model validation indicated that the model was highly significant.

### Differences in the metabolic pathways between the rat model and DVT patients

**Figure 8:**
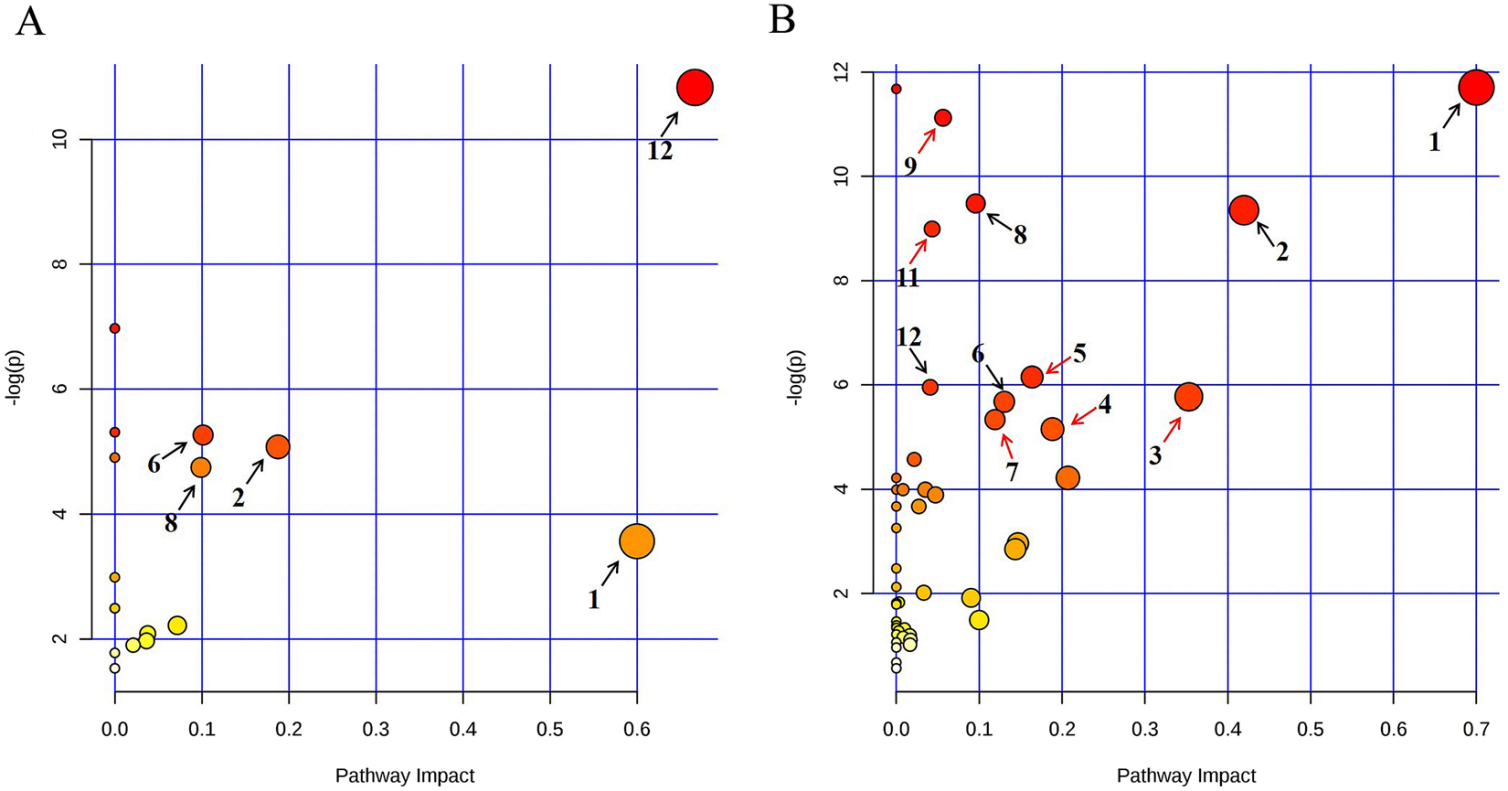
View map of the metabolic pathways. (**A**) The metabolic pathways of differential metabolites identified in rats with a DVT. Numbers in the figures represent the significant pathways depicted in Table 4. (**B**) The metabolic pathways of different metabolites identified in DVT patients. Numbers in the figures represent the significant pathways depicted in Table 5. This figure displays all matched pathways as circles. The *x*-axis represents enriched pathways, and the *y*-axis represents the impact pathways. The color and size of each circle is based on its p-value and the pathway impact value, respectively. Black arrows represent the same pathways in rats and humans, and red arrows represent the pathways only in humans.

To compare the 11 metabolites in rats and 20 metabolites in DVT patients, a file containing the list of Kyoto Encyclopedia of Genes and Genomes (KEGG) IDs, fold changes, and p-values adjusted for multiple comparisons was loaded into Metscape, and compound networks were created to obtain an overview of all differentially produced metabolites in the DVT rat model and patients (shown in Figure S2). The resulting network of the DVT rat model consisted of five components, as shown in Figure S2A; the largest subnetwork contained four metabolites, which were related to pyruvate metabolism. The resulting network of DVT patients consisted of six components (Figure S2B). The largest subnetwork, which contained 13 metabolites, was related to glycine metabolism. The metabolome view shown in Fig. 8 visualizes all matched pathways according to p values from pathway enrichment analysis and pathway impact values from pathway topology analysis. Although a number of pathway may be observed in the plots, it appears clear that 5 biochemical pathways were most-involved in the DVT rat model (Table 4 and Fig. 8A) and 12 most-involved pathways were detected in patients (Table 5 and Fig. 8B). The pathways of valine, leucine, and isoleucine biosynthesis; synthesis and degradation of ketone bodies; pyruvate metabolism; butanoate metabolism; and glycolysis or gluconeogenesis were all affected in the rats and patients. The aminoacyl-tRNA biosynthetic pathway was noteworthy, as it is involved in translation of genetic information processing in patients. The function of aminoacyl-tRNA synthesis is to precisely match amino acids with tRNAs containing the corresponding anticodon. Amino acids, such as glycine, phenylalanine, glutamine, valine, lysine, and tyrosine, were enriched in this pathway.

**Table 4:** Pathway analysis results from different DVT rat serum metabolites.

No.	Pathway name	Total^a^	Hits^b^	p values	log(p)	Holm p^c^	Impact^d^
1	Valine, leucine and isoleucine biosynthesis	11	3	1.97E-05	10.833	0.00159	0.667
2	Synthesis and degradation of ketone bodies	5	1	0.028247	3.5668	1.00000	0.600
3	Pyruvate metabolism	22	2	0.006219	5.0801	0.47886	0.188
4	Butanoate metabolism	20	2	0.005145	5.2698	0.40128	0.101
5	Glycolysis or Gluconeogenesis	26	2	0.00865	4.7502	0.64873	0.099

^a^Total is the total number of compounds in the pathway.

^b^Hits represents the actually matched number of metabolites in one pathway.

^c^Holm p is the p value adjusted by Holm-Bonferroni method.

^d^Impact is the pathway impact value calculated from a pathway topology analysis.

**Table 5:** Pathway analysis results from the different metabolites in serum from patients with a DVT.

No.	Pathway name	Total	Hits	p values	log(p)	Holm p	Impact
1	^*^Synthesis and degradation of ketone bodies	6	3	8.22E-06	11.708	6.58E-06	0.700
2	^*^Pyruvate metabolism	32	4	8.68E-05	9.3517	6.60E-03	0.420
3	D-Glutamine and D-glutamate metabolism	11	2	0.003113	5.7722	0.22413	0.353
4	Glycine, serine and threonine metabolism	48	3	0.005765	5.1560	0.39775	0.188
5	Methane metabolism	34	3	0.002139	6.1474	0.15829	0.164
6	^*^Butanoate metabolism	40	3	0.003427	5.6760	0.24334	0.130
7	Phenylalanine metabolism	45	3	0.004801	5.3390	0.33606	0.119
8	^*^Glycolysis or Gluconeogenesis	31	4	7.63E-05	9.4802	0.00588	0.096
9	Aminoacyl-tRNA biosynthesis	75	6	1.47E-05	11.126	0.00115	0.056
10	Propanoate metabolism	35	4	0.000125	8.991	0.00934	0.043
11	Arginine and proline metabolism	77	4	0.002604	5.9506	0.19012	0.041
12	^*^Valine, leucine and isoleucine biosynthesis	27	2	0.018427	3.9939	1.00000	0.035

^*^Represents pathways significantly altered in DVT rats and patients.

## Discussion

DVT is a common multifactorial disease resulting from interactions between genetic and acquired risk factors^17^. Due to the complexity of the pathogenesis and diagnosis, novel diagnostic and prognostic biomarkers are needed for suspected patients as well as for early diagnosis and timely treatment. In this pilot study, a NMR spectroscopic method was applied for the first time to study the metabolic profiles and relative metabolic pathways in rats and patients with DVT. The results showed differences in metabolites and pathways between the rat model and the DVT patients, indicating the feasibility of metabolomics for discriminating DVT disease. Additionally, our approach showed that metabolomics is a powerful tool to understand the molecular mechanisms of unprovoked DVT and prevent or reverse disease progression.

The rat inferior vena cava (IVC) ligation model, which has been widely contrasted in previous studies, was used in this present study^18–20^. Eleven compounds changed significantly in the serum of rats with a DVT derived from metabolic profiling compared with the control and sham groups (Table 2). The changes in acetoacetate (ketone bodies) and pyruvate were similar to those observed in an acute PE pig model^21^. The increased concentration of these two metabolites indicates that ketone bodies and glucose metabolism could be associated with DVT, probably due to hypoxia-mediated changes and glucose metabolism, and the results may be useful for understanding and explaining the pathogenesis of DVT.

In the present study, we have for the first time characterized changes in small metabolites in serum samples from unprovoked DVT patients. In order to identify the metabolites directly related to DVT, we enrolled 61 patients with DVT, including 30 males and 31 females. The average age of the patients was 59.58 ± 16.04. And 61 age- and gender-matched healthy volunteers were enrolled in the experiment. Since both the patients and the healthy control population are from Chinese Han, the result of the experiment only reflects the status of this race. A total of 20 significantly perturbed compounds were discovered from metabolic profiling as potential biomarkers for diagnosing DVT (Table 3). Some compounds, such as lipids, valine, acetoacetate, pyruvate, glucose, and methanol, changed in similar directions in patient and rat sera. These results suggest that these metabolites are directly associated with pathological conditions. The other different metabolites that showed contradictory trends between human and rat serum may be related to race, diet, or genetic factors. 3-HB, lactate, acetoacetate, pyruvate, creatine, and phenylalanine, which were observed in our patients, also changed in an animal group after acute PE^21^.

Multivariate and ROC curve analyses were conducted to assess the NMR spectroscopic methods for an early diagnosis of DVT. Figure 6 shows that patients could be discriminated from healthy individuals by metabolomics. The ROC curve shown in Fig. 8 indicates that 20 metabolites had high accuracy for an early diagnosis of DVT. These results demonstrate that metabolomics is an ideal technique to explore and evaluate DVT in the clinic, similar to phenylketonuria, diabetes, and obesity^22,23^.

MetPA was performed using the online software MetaboAnalyst to analyze the biological functions of these identified potential metabolite biomarkers^24,25^. The pathway, enrichment, and pathway topology analyses helped identify the most relevant pathways involved in DVT. The significantly altered pathways in the rats with DVT mainly involved carbohydrate, lipid, and amino acid metabolism. The altered pathways in patients were associated with carbohydrate metabolism, lipid metabolism, amino acid metabolism, energy metabolism, and genetic information processing. The thrombus in the DVT rat model was artificially created by surgery; therefore, the changes in the metabolic pathways that occurred in the rats with DVT should be directly caused by the thrombosis. Consistent with the rat pathways, the metabolic pathways of valine, leucine, and isoleucine biosynthesis; synthesis and degradation of ketone bodies; pyruvate metabolism; butanoate metabolism; and glycolysis or gluconeogenesis, which changed in patients, may be immediately induced by a thrombosis.

An energy metabolism-related pathway (methane metabolism) and a genetic information processing related pathway (aminoacyl-tRNA biosynthesis) were identified in patients, but not in rats with DVT. Most notably, the aminoacyl-tRNA biosynthesis pathway is involved in translation for genetic information processing. Six amino acids, such as glycine, phenylalanine, glutamine, valine, lysine, and tyrosine, were enriched in this pathway from patients with DVT. Aminoacyl-tRNAs are essential substrates for translation and are pivotal in determining how the genetic code is interpreted as amino acids. Aminoacylated tRNAs are synthesized by 3′-esterification of tRNAs with the appropriate amino acids, and the reaction is catalyzed by a family of enzymes collectively known as aminoacyl-tRNA synthetases (ARSs)^26–28^. The increasing discovery of genetic mutations in human ARSs is considered an important determinant of disease etiology. Some studies have demonstrated that mutations in genes encoding ARSs play important roles in inherited neurological diseases, such as peripheral neuropathies, encephalopathy, and ataxia^27,29–32^. The occurrence of a DVT requires the presence of several genetic and acquired risk factors. According to our results, aminoacyl-tRNA biosynthesis and the enriched metabolites may play important roles as genetic factors in DVT. In future studies, we will consider the aminoacyl-tRNAs and the ARSs.

A strength of this study was the analysis of the serum metabolic signatures of rats and patients simultaneously based on ^1^H NMR metabolomics. Two sets of metabolites were found in rats and patients with DVT, respectively, and the 20 significantly different metabolites in patients could be candidate biomarkers for diagnosing DVT. By comparing the metabolomic profiles from the two species, we found the pathways altered by the thrombosis and the pathways that were related to genetics in the patients. These results provide a foundation and new strategies to identify the pathological mechanism and pharmacological targets of DVT.

## Materials and Methods

### Reagents

Deuterium oxide (D_2_O, 99.9%D) was purchased from Sigma-Aldrich (St. Louis, MO, USA). Sodium 3-trimethylsilyl-(2,2,3,3-d_4_)-1-propionate (TSP) was purchased from Cambridge Isotope Laboratories, Inc. (Miami, FL, USA). Acetonitrile, NaH_2_PO_4_·2H_2_O, and Na_2_HPO_4_·12H_2_O (all analytical grade) were obtained from Guangfu Chemical Reagent Co. Ltd. (Tianjin, China). Phosphate buffer containing 0.01% TSP and 100% D_2_O was prepared with Na_2_HPO_4_ and NaH_2_PO_4_ (0.2 M, pH = 7.4) and used as the solvent for the ^1^H NMR analysis of the sample extracts.

### Rat experimental protocol

Healthy adult Sprague–Dawley rats (n = 30; all males, 180–200 g; Permission for Laboratory Animal Use: SCXK-2012–0004) were purchased from the Laboratory Animal Center of Academy of Military Sciences PLA China (Beijing, China). The animals were housed individually in metabolic cages with free access to water and food. All animal experiments were performed in accordance with the applicable Chinese legislation and approved by the Ethics Committee of Shanxi Medical University.

The rats were randomly divided into three groups (n = 10 per group): a control group, a DVT group, and a sham group. According to our previous study^33^, the rats in the DVT group were anesthetized with 10% chloral hydrate. After exploring the IVC, all side branches were ligated. The IVC was tied down on or just below the left renal vein. A microvascular clamp was attached to the confluence of the iliac veins for 15 min. The skin was sutured, and penicillin powder was applied. Sham-operated rats received anesthesia for all surgical procedures, but without IVC ligation or clamping. The control group received no treatment.

The rats were anesthetized with 10% chloral hydrate 72 h after ligation, and blood samples were drawn from the abdominal aorta. The upper layer (serum) was prepared by centrifugation at 3,000 × g for 10 min, transferred to cryovials in aliquots of 1 mL, and stored at −80 °C until analysis.

### Rat serum sample preparation

The ^1^H NMR samples were prepared as described previously^14^. After thawing at 0 °C, 450 µL of serum from each sample was mixed with 350 µL of D_2_O as a field lock and then centrifuged (13,000 rpm, 10 min, at 4 °C) to remove the precipitate. A 600-µL aliquot of the supernatant was transferred to a 5-mm NMR tube for ^1^H NMR analysis. Chemical shifts were calibrated against the lactate-CH_3_ signal (1.33 ppm).

### Patient enrollment and sample collection

All control and patients with DVT provided informed consent prior to the collection of the sample. The protocols of the study were approved by the Ethics Committee of Shanxi Medical University and conducted according to the principles expressed in the Declaration of Helsinki. Written informed consent statements were acquired from all recruited participants.

Patients with their first episode of unprovoked DVT were recruited from hospitals in Taiyuan and Shanxi from December 2015 to December 2016. All patients were diagnosed with a distal DVT through a higher D-dimer level and compression ultrasound. The inclusion criteria were adults with a suspected first episode of DVT and unprovoked DVT, excluding the provoking factors. The following were regarded as provoking factors: recent surgical trauma (within 8 weeks before the event), acute medical condition (acute myocardial infarction, acute ischemic stroke, or major infectious disease), cancer, marked immobilization (bed rest >3 days, wheelchair patients, or long distance travel ≥ 4 hours within the last 14 days), pregnancy or puerperium, estrogen supplementation, or other potential provoking factors specifically described by a physician in the medical records (e.g., intravascular catheter). Sixty-one patients with a DVT who fulfilled the criteria and volunteers acting as the healthy control group underwent the same screening and were age- and sex-matched.

Blood for the serum preparations was drawn from an antecubital vein in the morning after a 12-h overnight fast and immediately collected in 5-mL vacuum blood collection tubes. The serum was obtained by centrifugation at 3,000 × g for 10 min. Each serum sample was divided into equal aliquots and stored at −80 °C prior to analysis.

### Human serum sample preparation

After thawing at 0 °C, 450 µL of serum was mixed with 900 µL of cold methanol and centrifuged at 13,000 rpm for 20 min at 4 °C to remove the protein. A 900-µL aliquot of the supernatant was dried in the SCIENTZ-1LS (Ningbo Scientz Biotechnology Co., Ningbo, China) frozen centrifugal concentrator, and the dried samples were mixed with 600-µL of phosphate buffer (0.2 M Na_2_HPO_4_/NaH_2_PO_4_, pH = 7.4) in D_2_O containing TSP (0.01%) to minimize chemical shift variations, which was centrifuged (13,000 rpm, 10 min, at 4 °C) to remove the precipitate. A 550-µL aliquot of the supernatant was transferred to a 5-mm NMR tube for ^1^H NMR analysis.

### Metabolic profiling data acquisition

The ^1^H NMR spectra of serum from the rats and humans were obtained using a Bruker 600-MHz AVANCE III NMR spectrometer (Bruker Biospin, Rheinstetten, Germany) operating at a ^1^H frequency of 600.13 MHz and a temperature of 298 K. A one-dimensional Carr-Purcell-Merboom-Gill (CPMG, RD-90-(τcp-180-tcp)-acquisition with water suppression and a total spin-spin relaxation delay of 320 ms was used to attenuate the broad signals from proteins and lipoproteins due to their short transverse relaxation time. The ^1^H NMR spectrum for each sample consisted of 64 scans requiring 5 min of acquisition time using the following parameters: spectral width = 12019.2 Hz, spectral size = 65536 points, pulse width (PW) = 30° (12.7 µs), and relaxation delay = 1.0 s. The FIDs were Fourier transformed with LB = 0.3 Hz.

All acquired ^1^H NMR spectra were manually phased, and the baseline was set using MestReNova software (Mestrelab Research, Santiago de Compostella, Spain). Each spectrum was then segmented at *δ* 0.004 intervals across a chemical shift of 0.5–8.50. The area for each segment was calculated, and the integral values contributed to an intensity distribution for the entire spectrum. One region (*δ* 4.7–5.2) was excluded prior to statistical analysis to remove any variation in water suppression efficiency. All remaining regions of the spectra were scaled to the total integrated area of the spectrum to reduce any significant concentration differences. To reduce significant concentration differences between the samples, the integral values from each spectrum were normalized to the sum of all integrals in a spectrum for further multivariate analysis. The chemicals shifts were referenced to the lactate signal^16^ (1.33 ppm) for the rat serum and the TSP signal^14^ (0 ppm) for the patient serum samples.

### Data processing and statistical analysis

All resulting integral data from the ^1^H NMR metabolomic analysis were introduced into SIMCA-P 14.1 (Umetrics, Malmö, Sweden) for the multivariate analysis. Initially, a PLS-DA was performed to distribute and separate the different groups in a supervised manner. The quality of the model was described by the parameters for model fitness (R^2^) and predictive ability (Q^2^), where a large R^2^ (close to 1) and Q^2^ (Q^2^ ≥ 0.5) indicated a good model. Next, the PLS-DA model was validated by the response values of the permutation test in which the class membership was randomly shuffled 200 times. The results indicated a lack of over-fitting when the new R^2^ and Q^2^ values were lower than the original values. Additionally, another supervised pattern recognition approach using orthogonal projection to latent structures discriminant analysis (OPLS-DA) was performed to improve the classification of the different groups, as well as to screen for biomarkers. This method excavates variables from complex data sets to identify metabolites for distinguishing the two groups. Additionally, a p-value (p-value < 0.05) from a CV-ANOVA was used to indicate the level of significance for group separation in the OPLS-DA based on the cross-validated model. To further understand the potential variables contributing to the differences, we performed an S-plot analysis with the OPLS-DA model, where each coordinate reflected the NMR spectral region (metabolite signal), which was used to define the metabolites that significantly contributed to separate the group. The key metabolites that were necessary for distinguishing between groups were selected from the results of the VIP value for the established OPLS-DA model analysis (VIP > 1).

Furthermore, Student’s *t*-test was used to evaluate the differences in the selected signals of the main metabolites that were responsible for class discrimination using SPSS 16.0 (SPSS Inc., Chicago, IL, USA). ROC curves and the AUC values were determined to further evaluate the performance of the metabolites from DVT patients in the clinical diagnosis. The metabolites were entered into Metscape, a metabolic network analysis and visualization tool, to generate the networks associated with each of the different metabolites^34^. The metabolites that were significantly different (p < 0.05) in serum samples between the DVT and controls from rats and humans were analyzed using the pathway topology search tool in MetaboAnalyst 3.0 (MetPA). The pathway libraries chosen were for *Homo sapiens* (human) and *Rattus norvegicus* (rat). Fisher’s exact test was applied to perform an over-representation analysis, and relative betweenness centrality was chosen for pathway topology testing. Pathways with a raw p < 0.05 were considered altered due to DVT.

## Electronic supplementary material

Supplementary Materials
