# Supplementary material for: Comparison of the serum metabolic signatures based on 1H NMR between patients and a rat model of deep vein thrombosis
Source: Sci Rep. 2018 May 18;8:7837. doi: 10.1038/s41598-018-26124-x (PMC5959905; doi:10.1038/s41598-018-26124-x)
Supplement: Supplementary file 1 — Supplementary Materials [file 41598_2018_26124_MOESM1_ESM.pdf]

Comparison of the serum metabolic signatures based on ^{1}H NMR between patients and a rat model of deep vein thrombosis

Jie Cao[1], Qian-qian Jin[1], Gui-ming Wang[2], Hong-lin Dong[3], Yong-ming Feng[1], Jun-sheng Tian[4], Ke-ming Yun[1], Ying-yuan Wang[1]*, and Jun-hong Sun[1]*

[1]School of Forensic Medicine, Shanxi Medical University, Taiyuan 030001, P.R. China.

[2]Department of Vascular Surgerey, First Hospital of Shanxi Medical University, Taiyuan 030001, P.R. China.

[3]Department of Vascular Surgerey, Second Hospital of Shanxi Medical University, Taiyuan 030009, P.R. China.

[4]Modern Research Center for Traditional Chinese Medicine, Shanxi University, Taiyuan 030006, P.R.China.

*Corresponding and requests for materials should be addressed to Jun-hong Sun (email: junhong.sun@sxmu.edu.cn) and Ying-yuan Wang (wyy580218@163.com); Tel : +86-351-4135175 ; Fax : +86-351-4135197.

Address : School of Forensic Medicine, Shanxi Medical University, 56 South Xinjian Road, Taiyuan 030001, Shanxi, PR China

Contents

Supplementary Figures

Figure S1. Receiver operating characteristic (ROC) curve analysis of the 20 metabolites.

Figure S2. Bioinformatics analysis of the different metabolites.

Supplementary Figures

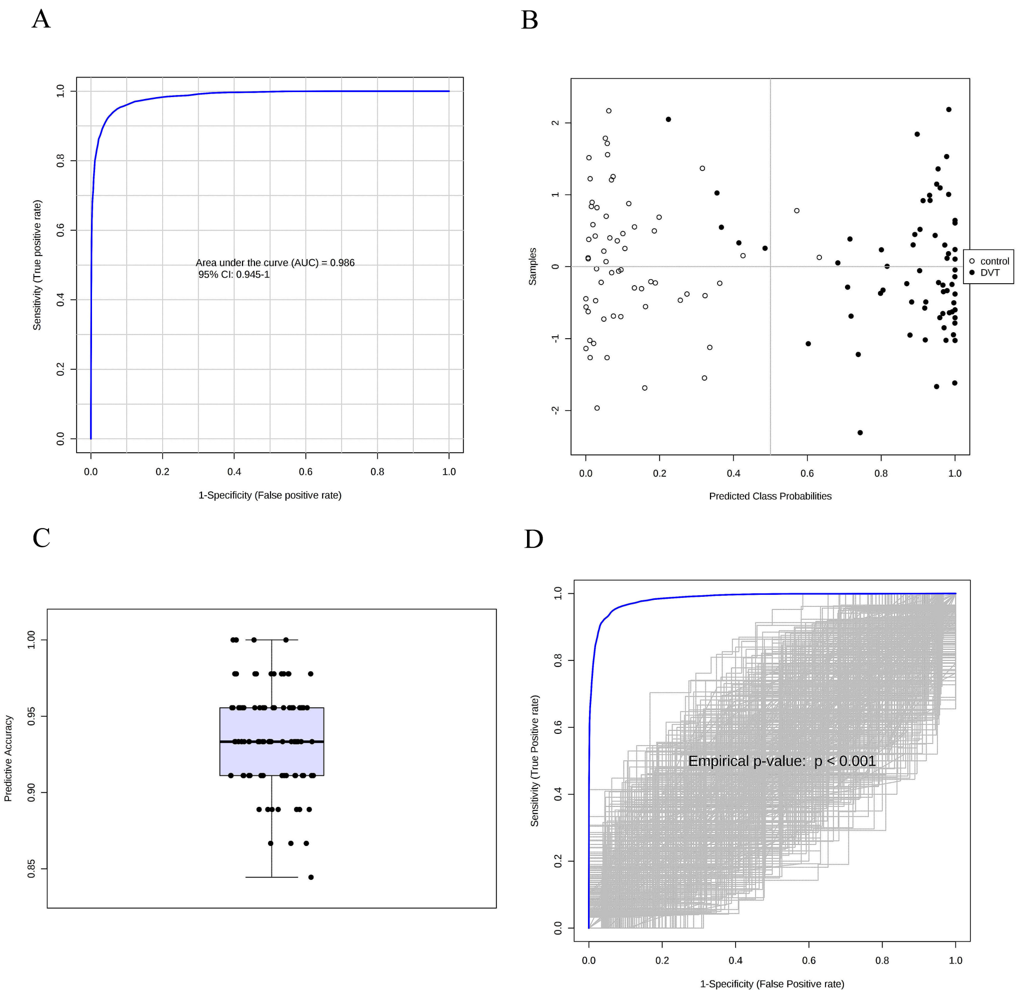

Figure S1. Receiver operating characteristic (ROC) curve analysis of the 20 metabolites. (A) The ROC curve of the biomarker model. (B) The predicted class probabilities of all samples using the biomarker model. (C) Box plot of the predictive accuracy of the biomarker model. (D) The permutation tests (n = 1,000) for the biomarker model.

A

B

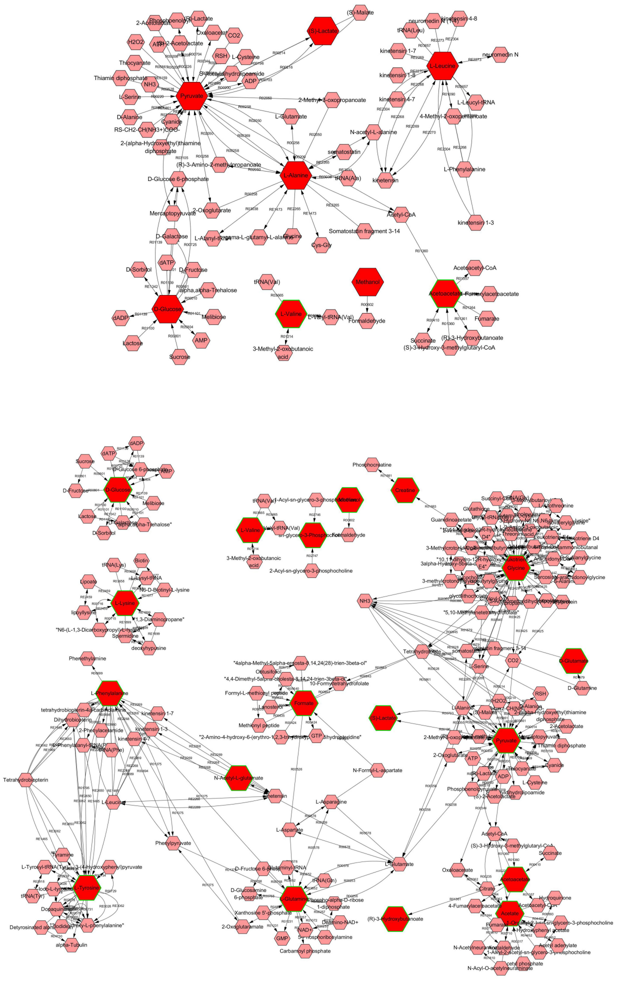

Figure S2. Bioinformatics analysis of the different metabolites. (A) The network of different metabolites identified in rats with a DVT generated by Metscape. (B) The network of different metabolites identified in patients with a DVT. Hexagons represent the compounds in the network, red represents metabolites with experimental data, and light red hexagons are the compounds that participate in the networks.
